# Modeling and Optimization of Structural Parameters for High-Efficiency Multi-Jet Polishing of Optical Glass

**DOI:** 10.3390/mi16050551

**Published:** 2025-04-30

**Authors:** Zhongchen Cao, Yiwei Miao, Ming Wang, Zhenfeng Zhu

**Affiliations:** Key Laboratory of Mechanism Theory and Equipment Design of Ministry of Education, Tianjin University, Tianjin 300072, China; zhongchen_cao@tju.edu.cn (Z.C.); yiweimiao0203@163.com (Y.M.); wang17th@outlook.com (M.W.)

**Keywords:** multi-jet polishing (MJP), computational fluid dynamics (CFD), modeling, structural parameters, optical glass

## Abstract

Multi-jet polishing (MJP) is a promising method for enhanced polishing efficiency by integrating multiple nozzles, allowing for the high-efficiency polishing of large-scale surfaces. However, the optimization of the structural parameters, such as the distribution form of the nozzles and outlet diameter, remains a critical challenge for achieving uniform and stable polishing performance. This paper presents a dynamic model of MJP based on the theory of fluid dynamic pressure and particle erosion. The flow field and particle motion characteristics in multi-nozzle jet polishing were studied using simulation experiments. The influence of the nozzle spacing and form and outlet diameter on the flow field characteristics and material removal profile was explored, and the structural parameters of the multi-nozzle polishing tool were optimized. According to the simulation results, two kinds of multi-nozzle polishing tools with a linear arrangement and cross arrangement were processed, and a series of single-point and surface polishing experiments was carried out. The optimized multi-nozzle jet polishing tool has no interference in the removal contour of each point, exhibits high consistency and stability, and is consistent with the theoretical model prediction results, which effectively improve the surface polishing efficiency. The results can provide a theoretical and experimental reference for MJP in the ultra-precision and high-efficiency polishing of large-sized components.

## 1. Introduction

Optical glass components have been widely used in the aerospace, semiconductor, and energy industries because of their excellent electrical insulation, high strength, and low thermal expansion coefficient [1]. To meet the surface requirements of optical glass components, ultra-precision machining technology is generally required to achieve nanometer-level surface roughness and high surface shape accuracy [2,3,4]. However, the brittleness and structural complexity of optical glass components lead to mechanical fracture and interference, which pose a great challenge in precision optical manufacturing [5]. Fluid jet polishing (FJP) [6,7] is an effective method for finishing complex optical surfaces because of its localized pressure in the jet beam, stable and controllable material removal function, low cost, lack of thermal damage, and high adaptability [8,9,10]. To further improve the polishing efficiency, scholars proposed multi-jet polishing (MJP), which uses a specific array of nozzles to simultaneously finish the workpiece from multiple nozzle regions [11,12]. The interference between the jet beams affects the uniformity and accuracy of the material removal [13]. Therefore, in-depth research on the interaction mechanism of multi-nozzle jets and the optimization theory of nozzle structures is of great importance to promote the development and application of MJP.

Research has been conducted to investigate the effect of the multi-nozzle array structure on the polishing efficiency and precision of MJP. Luo et al. [14] first proposed the multi-nozzle structure and found that the removal rates of the three-nozzle and seven-nozzle structures were significantly higher than that of a single nozzle; this study confirmed the theoretical feasibility of the multi-nozzle structure for improving the processing efficiency. Qiao et al. [15,16] further demonstrated the influence of the nozzle outlet shape on the uniformity and removal efficiency during material removal. The array-hole nozzles outperformed array-slit nozzles, with the machining efficiency of the nozzles showing a positive correlation with the outlet area. However, these studies initially explored a nozzle structure with an equidistant staggered arrangement or the effects of the nozzle shape and quantity without considering other relevant parameters. In terms of the process and application, Wang et al. [11,17] verified the feasibility of MJP using a CFD simulation and proposed curvature-adaptive multi-jet polishing (CAMJP), in which each nozzle is equipped with an independent pressure control system to achieve precise control. In addition, Cheung et al. [18] designed a slender rod-shaped nozzle with a linear array of orifices at its side face for polishing internal cylindrical surfaces to enrich the application field of MJP. The current research mainly focuses on optimizing the number and shape of nozzles. Few theoretical and experimental studies have been conducted on the influence of the nozzle diameter, spacing, and arrangement on flow field interference, and these parameters need to be further improved.

Given the lack of theoretical guidance for the design of the current multi-nozzle structure, we established a computational fluid dynamics (CFD) model to elucidate the characteristics of the flow field and particle erosion in MJP. The influence of the nozzle spacing, diameter, and distribution on the flow field characteristics and the corresponding material removal characteristics were investigated. The results provide theoretical guidance for the design optimization of multi-nozzle tools. Furthermore, the validity of the numerical simulation model was verified through a series of single-point experiments. The multi-nozzle structure was optimized and fabricated according to the simulation results, and a surface polishing experiment was carried out. This work provides theoretical and experimental guidance for the development of multi-nozzle jet polishing technology.

## 2. Theoretical Modeling

This section presents the modelling process and boundary condition settings of the MJP simulation model and analyzes the computational fluid dynamics theory involved in the numerical simulation, as well as the single-abrasive particle erosion removal model. First, a 3D model of the multi-nozzle is established with meshing and boundary conditions. The volume of fluid (VOF) model and the realizable k-ε model are used to describe the multiphase flow and turbulence characteristics. The motion trajectory of abrasive particles is simulated using the discrete phase model (DPM), and the particle rebound model is introduced to analyze the particle velocity. Finally, an erosion model combined with fractal theory is developed to predict the material removal and surface roughness to supply a theoretical basis for the optimization of MJP.

### 2.1. Geometric Modeling

**Figure 1:**
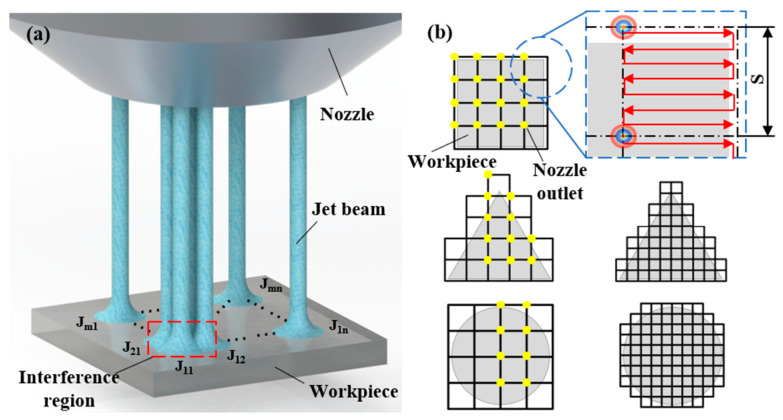
Schematic diagram of the principle of MJP: (**a**) multi-nozzle array structure; (**b**) layout of nozzles with different workpiece shapes.

**Figure 2:**
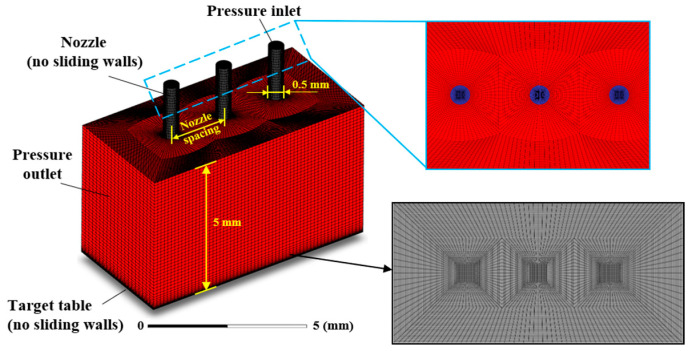
Mesh diagram of the multi-nozzle structure.

To simulate and analyze MJP under different multi-nozzle structures, a 3D simulation model is built. Figure 1a provides a schematic representation of the underlying principle of MJP. The efficiency of jet polishing can be markedly enhanced by designing multi-nozzle polishing tools with varying patterns and spacing to avoid any deterioration in surface quality. Figure 1b shows the nozzle outlet layout scheme for polishing workpieces of different shapes by using multiple nozzles. Figure 2 illustrates the mesh schematic of the multi-nozzle structure and the boundary conditions with linear distributed nozzles as an example. Table 1 lists the complex model parameter configurations employed throughout the simulation, where the target distance in MJP is 5 mm and the nozzle spacing indicates the distance between the axes of neighboring nozzle outlets. In this simulation, the flow field characteristics are analyzed for different nozzle spacings, diameters, and distributions. Based on the FJP flow field characteristics, a discrete phase approach is used to elucidate the kinematic properties of the abrasive particles. Simulations are conducted using cerium oxide (CeO_2_) abrasive particles with a mass density of 7130 kg/m^3^ and a particle diameter of 6 µm.

### 2.2. Continuity Equation for the Continuous Phase

The VOF model is employed to describe the multiphase flow in jet polishing [19], with air as the main phase and the polishing slurry as a secondary term, when interfaces between multiple immiscible fluids are required.

When MJP is performed, the mutual collision between the jet bundles produces a significant pressure gradient, as well as vortex features in the interference region, which exacerbates the turbulence of the fluid. The realizable *k*-*ε* turbulence model has significant computational advantages in solving vortex shear flow, free flow, and boundary layer flow [20,21,22]; as such, the realizable *k*-*ε* model is chosen to describe the turbulence phenomenon in the complex flow field of a polished multi-nozzle jet with the following transport equations:

(1) $$\frac{\partial }{\partial t}\left(\rho k\right)+\nabla \left(\rho kv\right)=\nabla \left[\left(\mu +\frac{\mu_{t}}{\sigma_{k}}\right)\nabla k\right]+G_{k}+G_{b}-\rho \varepsilon$$

(2) $$\frac{\partial }{\partial t}\left(\rho \varepsilon \right)+\nabla \left(\rho \varepsilon v\right)=\nabla \left[\left(\mu +\frac{\mu_{t}}{\sigma_{\varepsilon }}\right)\nabla \varepsilon \right]+\rho C_{1}\varepsilon S-\rho C_{2}\frac{\varepsilon^{2}}{k+\sqrt{v\varepsilon }}+G_{1\varepsilon }\frac{\varepsilon }{k}G_{b}G_{3\varepsilon }$$

where k denotes turbulent kinetic energy; ε denotes the turbulent dissipation rate; $\mu_{t}$ denotes the turbulent viscosity coefficient; $G_{k}$ and $G_{b}$ are the mean velocity gradient and lift-induced turbulent kinetic energy, respectively; $\sigma_{k}$ and $\sigma_{\varepsilon }$ denote the Prandtl numbers of k and ε, respectively; $G_{1\varepsilon }$, $G_{3\varepsilon }$, and $C_{2}$ are empirical constants, with values of 1.44, 1, and 1.9, respectively [23]. In addition, the following equation is used to calculate $C_{1}$ [24]:

(3) $$\left\{\begin{array}{c} C_{1}=\max\left[0.43,\frac{\eta }{\eta +5}\right]\\ \eta =S\frac{k}{\varepsilon }\\ S=\sqrt{2E_{s}\cdot E_{s}} \end{array}\right.$$

where $E_{s}$ is the modulus of the strain rate tensor averaged over time.

### 2.3. Discrete Phase Governing Equations

A discrete phase model (DPM) is employed for simulation to gain insights into the trajectory of abrasive particles in MJP. The concentration of the jet polishing slurry is usually lower than 10%, so the collision between particles can be ignored, and they are regarded as Lagrangian particles. When the flow field is turbulent, a discrete wandering stochastic model is used, considering the influence of turbulence on particle diffusion. According to Newton’s second law, the particle mass is $m_{p}$, and the mechanical equilibrium equation of a single particle is:

(4) $$m_{p}\frac{dv_{p}}{dt}=F_{D}+\frac{m_{p}g\left(\rho_{p}-\rho \right)}{\rho_{p}}+F_{a}$$

where $v_{p}$ represents the velocity of the particle; $F_{D}$ represents the fluid trailing drag force on the particle; $\rho_{p}$ is the particle density; the second term on the right-hand side of the equation is the gravity force on the particle; $F_{a}$ is the additional force on the particle, including Brownian force, virtual mass force, and Saffman lift force. The trailing force $F_{D}$ can be expressed as [25]:

(5) FD=mp18μρpdp2CDRe24v−vp

where $d_{p}$ refers to the diameter of the abrasive particle; Re is the relative Reynolds number; and $C_{D}$ is the trailing force coefficient, which is mainly affected by the shape of the abrasive particles. In this work, the experiments use polygonal abrasive particles, which are not spheres, so the shape factor is modified to 0.85 [26,27].

### 2.4. Particle Rebound Model

When particles diffuse in the jet system, collision rebound may occur with the wall. The recovery ratio parameter is adopted to measure the trajectory and momentum lost by the particles when impacting the wall to analyze the velocity change of the particles [28]. The formula is given by:

(6) $$V_{N_{2}}/V_{N_{1}}=0.993-0.0307\beta +0.000475\beta^{2}-2.61E^{-6}\beta^{3}$$

(7) $$V_{T_{2}}/V_{T_{1}}=0.988-0.029\beta +0.000643\beta^{2}-3.56E^{-6}\beta^{3}$$

where $V_{N_{1}}$ and $V_{N_{2}}$ are the normal velocities of the particles before and after impacting the wall; $V_{T_{1}}$ and $V_{T_{2}}$ are the tangential velocities of the particle before and after impacting the wall; β is the angle (°) at which the abrasive particle hits the wall.

### 2.5. Abrasive Particle Erosion Theory

Jet polishing can remove optically hard and brittle materials in a completely plastic manner without creating new damage. For MJP, the interference between the jets does not alter the erosion mechanism of the abrasive particles. However, it does create a complex flow field on the workpiece surface. Cao et al. [29] proposed a ductile-mode erosion model for predicting 3D material removal characteristics. In this paper, Cao’s model is further combined with the above simulation results of flow field and particle motion characteristics. The spatial distribution of particles is influenced by the abrasive concentration c, the slurry velocity u, and the impact position x, which can be described by:

(8) $$\begin{array}{c} N(c,u,x)=k_{\eta }k_{n}\times cut\pi {\left(\frac{d_{n}}{2}\right)}^{2}\times \frac{6}{\rho_{p}\pi d_{p}^{3}}\times \exp\left(-\frac{1}{2}{\left|\frac{x-C}{B}\right|}^{\lambda }\right)\\ =\frac{3k_{\eta }k_{n}cutd_{n}^{2}}{2\rho_{p}d_{p}^{3}}\exp\left(-\frac{1}{2}{\left|\frac{x}{B}\right|}^{\lambda }\right) \end{array}$$

where $k_{\eta }$ and $k_{n}$ are the efficiency factor and scale factor, respectively; cut refers to the particle spatial distribution at the inlet; $d_{n}$ is the nozzle diameter, and $\rho_{p}$ and $d_{p}$ are the density and diameter of the particles, respectively; x is the position variable; C indicates the position of the curve’s peak; B controls the width of the curve; λ influences the shape of the curve.

The total material removal rate when a single abrasive particle impacts the surface of the sample can be understood as the sum of the material volumes removed by the abrasive particle in the normal component and the horizontal component; that is:

(9) $$\begin{array}{c} \Delta E_{s}=\Delta E_{h}+\Delta E_{v}\\ =C_{1}\frac{m_{p}^{1+2\left(1-b\right)/3}V_{0}^{2+4\left(1-b\right)/3}{\left(\cos\delta \right)}^{2}{\left(\sin\delta \right)}^{4\left(1-b\right)/3}}{P_{t}P_{n}^{2\left(1-b\right)/3}{\left(\tan\phi \right)}^{\left(1-b\right)/3}}\\ +C_{2}\frac{m_{p}^{1+n}V_{0}^{2\left(1+n\right)}\sin^{2\left(1+n\right)}\delta }{P_{n}} \end{array}$$

where $\Delta E_{h}$ represents the volume of material removed along the horizontal axis; $\Delta E_{v}$ represents the volume of material removed along the vertical axis; $C_{1}$, $C_{2}$, b, and n are the fitting coefficients; $m_{p}$ represents the particle mass; δ represents the impact angle; $V_{0}$ represents the particle impact speed; ϕ represents the half-angle of the conical particles; and $P_{t}$ and $P_{n}$ are the pressure associated with plastic flow.

The volume of material removed by a single abrasive particle is influenced by several factors, including the particle’s mass, size, impact angle, velocity, and material properties, as well as the roughness of the target surface. According to Goodwin and Huang, this volume correlates exponentially with the velocity, typically within the range of 2 to 2.3 [30,31]. In this study, the velocity exponent is set to 2.3, leading to an index of b≈0.3 and n≈0.8. Experimental methods can also be used to determine these coefficients. Based on the studies and preliminary experiments of Huang and Cao, the coefficients $k_{1}$ and $k_{2}$ are found to be $k_{1}=0.82$ and $k_{2}=3.5\times 10^{-3}$ [29]. As the abrasive particles are mainly driven by the fluid to impact the sample surface, the normal $P_{t}$ and tangential $P_{n}$ pressures calculated via the CFD model for particle impact can approximate the fluid pressure.

## 3. Numerical Simulation and Experimental Analysis

### 3.1. Numerical Simulation

This part uses the CFD model to analyze the complex flow field characteristics of the multi-nozzle structure and the particle erosion characteristics. The removal profiles for jet polishing under different conditions are calculated by combining the simulation results with the single abrasive erosion removal model. The results provide theoretical guidance for the optimization of the multi-nozzle structure design.

#### 3.1.1. Effect of Nozzle Spacing

**Figure 3:**
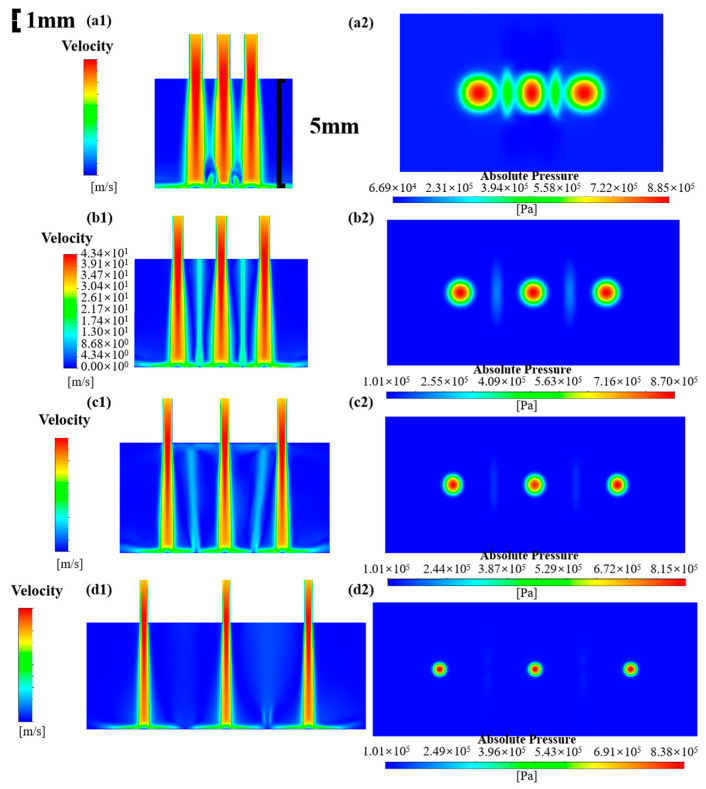
Flow field distribution and workpiece surface pressure at different nozzle spacings: (**a1**,**a2**) 1 mm; (**b1**,**b2**) 2 mm; (**c1**,**c2**) 3 mm; (**d1**,**d2**) 5 mm.

In this subsection, the simulation of the multi-nozzle structure, with a nozzle diameter of 0.5 mm and spacings of 1, 2, 3, and 5 mm, is carried out to analyze the flow field distribution and particle erosion signatures. The fluid velocity field and the pressure distribution on the impinged wall are displayed in Figure 3. It is a fact that when multiple jets impact the workpiece and the nozzle spacing is small, a low-pressure region is formed around the high-speed core of the jets, which leads to the intersection of the jets between neighboring nozzles to form an interfering beam of jets, which affects the stability of the multiple-nozzle jet beam and the stability of the removal function. Figure 3(a1) presents the velocity distribution of the nozzle with 1 mm spacing. The interference is very severe, and the jet beams gradually disperse and intermingle with each other before reaching the target surface. For a single-nozzle polishing tool, the jet beam impacts the workpiece surface and then spreads out in all directions. As the fluid increases its distance from the center of impact, the energy of the jet beam is gradually consumed and eventually flows out of the processing area. Upon impact with the workpiece surface, the jet beam undergoes a sudden change in direction within the central region, resulting in the formation of a high-pressure stagnation zone characterized by a region of almost zero internal flow velocity [32]. This phenomenon results in difficulty of the abrasive particles in traversing the region. However, in response to the fluid traction exerted by the flow along the direction of movement towards the stagnation zone, a ring-shaped contour of material removal is ultimately formed. As for the multi-nozzle structure, adjacent beams collide with each other in the process of spreading in all directions. The resulting liquid splash further interferes with the stability of the beams. In addition to the stagnation zone caused by the three jet beams, the fluid velocity is almost zero in the interference region, indicating that a stagnation zone is also generated at the location of the collision of the jet beams. This phenomenon is also well reflected in the impact wall pressure distribution in Figure 3(a2). The three red areas in the figure are the stagnation zones generated by the three jet beams, as well as two “shuttle” high-pressure areas in their intermediate positions, which show the distribution of two sharp ends and a wide middle. Compared with the stagnation zone generated by the jet beam itself, the stagnation pressure generated by the interference is obviously lower but the changes in the pressure and velocity distribution of the flow field will inevitably change the removal characteristics of the jet polishing material. In addition, the stagnation zone corresponding to the nozzle outlet in the middle is compressed by the jet beam on both sides, and the pressure distribution shows an elliptical shape. An increase in the nozzle outlet spacing to 2 mm eliminates the staggering phenomenon of the jet beams prior to their impact on the surface of the workpiece. However, the interference between the jet beams after the impact persists, albeit with reductions in both the area of the interference zone and the value of the pressure. The pressure distribution in the middle of the stagnation zone is restored to a circular shape, as illustrated in the Figure 3(b1,b2). With the increase in the nozzle outlet spacing, the interference gradually decreases. When the spacing increases to 5 mm, the interference area basically disappears, indicating that the jet beams are significantly reduced after diffusion of the impact energy and cannot form an effective interference effect, as shown in Figure 3(d1,d2). In summary, the jet beam interference is serious when the nozzle spacing is 1 mm; moreover, the interference is reduced, and the stability of the beams is improved with increasing spacing. An increase in the spacing to 5 mm results in the complete elimination of interference.

**Figure 4:**
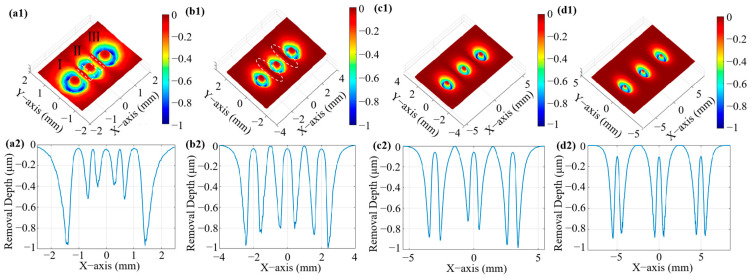
Three-dimensional simulation of removal function and cross-sectional profiles at different spacings: (**a1**,**a2**) 1 mm; (**b1**,**b2**) 2 mm; (**c1**,**c2**) 3 mm; and (**d1**,**d2**) 5 mm. The labeled contours I, II, and III in (a1) correspond to the removal profiles of the outer left, central, and outer right nozzles, respectively.

Based on the simulation results of the flow field characteristics, the material removal rate of the multi-nozzle polishing tool is normalized. The simulation optimization focuses mainly on the region where material removal occurs rather than the specific removal depth in the case of significant jet beam interference. Figure 4 illustrates the distribution of the removal rates and removal depths predicted by the simulation model for different nozzle outlet spacings. As shown in Figure 4(a1), the removal contours corresponding to the three nozzle outlets are labelled, with serial numbers I and III indicating the removal contours corresponding to the two outer nozzles, and serial number II indicating the removal contour corresponding to the nozzle in the center position. When the nozzle spacing is 1 mm, the removal function at II is severely deformed, and the annular removal contour becomes elliptical; at the same time, the two removal contours at I and III also become incomplete due to the interference problem between the jet beams. This finding also corresponds to the removal depth curves in Figure 4(a2). The removal depths at I and III are large; the jet beams at II are squeezed by the interference between the two jet beams, and the removal depths are reduced, especially in the area of the white box in Figure 4(a1). When the nozzle outlet spacing is increased to 2 mm, as shown in Figure 4(b1,b2), the degree of deformation of the removal profile is improved and the inhomogeneity of the removal depth is reduced; however, the interference phenomenon still exists, and additional material removal due to the fluid interference problem can be observed as elongated shapes in the white dashed area in Figure 4(b1). After further increasing the distance to 3 mm, as shown in Figure 4(c1,c2), the removal profile caused by each jet beam of the multi-nozzle polishing tool is similar to the single-nozzle annular removal effect, which causes a homogeneous distribution of the removal depth, and the interference phenomenon is further reduced. When the nozzle outlet spacing reaches 5 mm, as shown in Figure 4(d1,d2), three removal profiles with the same diameter, depth, and shape can be observed on the machined surface, although interference does not exist, with the removal profiles being close to those of the single-nozzles, showing the optimum removal effect. As the nozzle outlet spacing increases, the removal becomes more uniform and consistent, and the interference decreases. In summary, for a multi-nozzle jet polishing tool with a nozzle outlet diameter of 0.5 mm, the nozzle outlet spacing should be no less than 5 mm.

#### 3.1.2. Effect of Outlet Diameter

**Figure 5:**
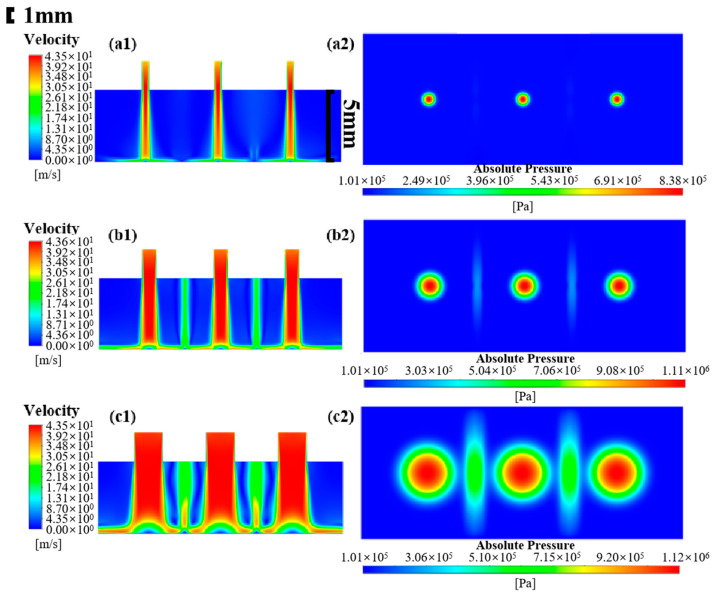
Flow field distribution and workpiece surface pressure of multiple nozzles with different nozzle diameters: (**a1**,**a2**) 0.5 mm; (**b1**,**b2**) 1.0 mm; and (**c1**,**c2**) 2.0 mm.

In this subsection, the particle erosion characteristics and flow field characteristics are analyzed using a computational fluid dynamics simulation model for nozzle diameters of 2.0, 1.0, and 0.5 mm, respectively, with the nozzle outlet spacing fixed at 5 mm and the rest of the process parameters kept the same. Figure 5(a1,a2) shows the fluid velocity field and the pressure distribution on the impacted wall when the nozzle diameter is 0.5 mm. The interference basically disappears, and no obvious high-pressure distribution can be observed in the region between adjacent nozzles. In contrast, as the diameter is increased to 1.0 mm, the interference between the jet beams is serious, and the fluid shock phenomenon is obvious in the region between the adjacent jet beams, as shown in Figure 5(b1). Figure 5(b2) shows its pressure distribution cloud, and we can see that as the nozzle diameter increases, the region of the high-pressure stagnation zone increases; at the same time, a high-pressure region is generated at the location where the collision of the jet beams occurs. When the nozzle diameter is 2.0 mm, the jet beam diameter increases, the velocity of the upward impinging jet due to the beam interference increases significantly, and the area of the high-pressure region in the beam interference area and the pressure value increase significantly, as illustrated in Figure 5(c1,c2).

**Figure 6:**
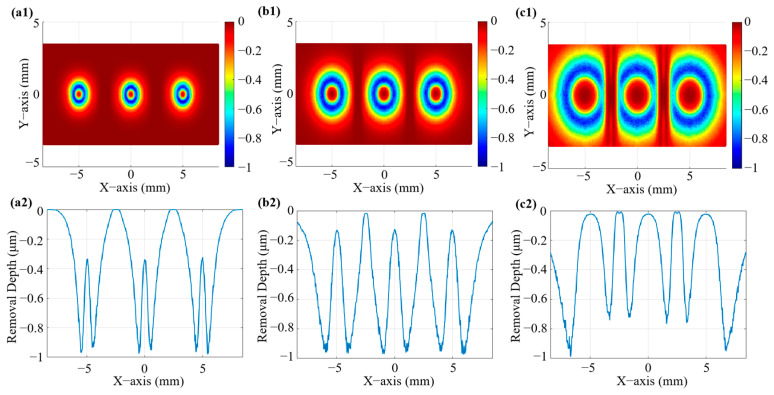
Three-dimensional simulation of removal function and cross-sectional profiles at nozzle diameters: (**a1**,**a2**) 0.5 mm; (**b1**,**b2**) 1.0 mm; and (**c1**,**c2**) 2.0 mm.

Figure 6 shows the distributions of the material removal rate and removal depth at different nozzle diameters. As the nozzle diameter increases, the diameter and removal depth of the removal contour gradually increase but the interference between the removal contours also increases. As such, the inhomogeneity of the removal depth distribution increases, which is caused by the increase in the area of the stagnation zone. Specifically, under the 0.5 mm nozzle diameter, the removal rate and removal depth distribution are uniform, and the interference phenomenon is not obvious, as shown in Figure 6(a1,a2). Under the 1.0 mm nozzle diameter, the deformation of the removal contour and the inhomogeneity of the removal depth distribution begin to appear, as shown in Figure 6(b1,b2). Under the 2.0 mm nozzle diameter, the interference phenomenon is more obvious, the removal contour is significantly deformed, the removal function at the middle position becomes elliptical, and the inhomogeneity of the removal depth distribution further increases, as shown in Figure 6(c1,c2). The above simulation results show that for MJP tools, the spacing between the nozzles needs to be optimized according to the nozzle diameter. It is essential to minimize the interference between the jet beams and ensure the attainment of the requisite quality and uniformity for polishing.

#### 3.1.3. Effect of Distribution

**Figure 7:**
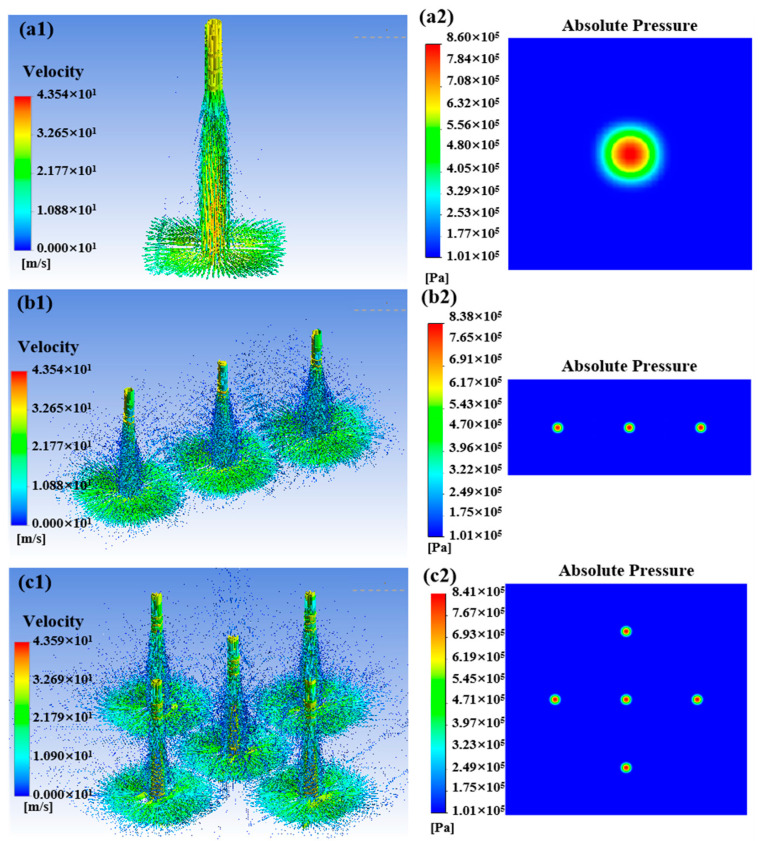
Particle erosion trajectories and workpiece surface pressure under different distribution forms: (**a1**,**a2**) single nozzle; (**b1**,**b2**) linear distribution; and (**c1**,**c2**) cross.

Under the condition of a nozzle diameter of 0.5 mm and outlet spacing of 5 mm, this subsection simulates the flow field and particle erosion characteristics under different nozzle outlet distributions. Figure 7 illustrates the fluid velocity field and the pressure distribution of the impinged wall under the distribution of a single nozzle, a straight line with three nozzles, and a cross-crossing distribution, respectively, which shows that the interference between the jet beams is complicated by the increase in the number of nozzle outlets and the diversification of the distributions. With the increase in the number of nozzle outlets and the diversification of the distribution, the interference between the jet beams is complicated. In Figure 7(a1,a2), a single jet beam impinges on the workpiece surface and then disperses uniformly in all directions without interference from other flow fields. In Figure 7(b1,b2), when the number of nozzles is increased to three, the center jet beam is interfered with by the flow fields on both sides. When the nozzle outlet distribution is set as a cross-cross distribution, the jet beam in the middle position is interfered with by the four adjacent direction jets, and upward splashing occurs due to mutual collision of fluids at the junction position. However, no change in pressure distribution due to the interference of jet beams can be observed in the pressure distribution diagram of the impingement wall. Five independent high-pressure regions can still be observed, as shown in Figure 7(c1,c2). Although the flow field is complex due to the criss-crossing of the nozzle outlets, the 5 mm nozzle outlet spacing ensures a lack of serious interference between the jet beams on the velocity distribution and the wall pressure distribution; consequently, the material removal effects are independent of each other.

### 3.2. Experiment

This section presents the findings of two-stage experiments, which comprise spot polishing and surface polishing experiments. First, a new multi-nozzle polishing tool head was designed based on the optimization results of the complex flow field simulation. The effectiveness and accuracy of the multi-nozzle complex flow field simulation and the removal profile prediction model were verified through spot polishing experiments. This work investigated the efficiency of the multi-nozzle polishing tool design. Single-nozzle and multi-nozzle tool head surface polishing comparison experiments were conducted to measure the processing efficiency by comparing the quality changes under the same process parameters and processing time.

#### 3.2.1. Experimental Design

**Figure 8:**
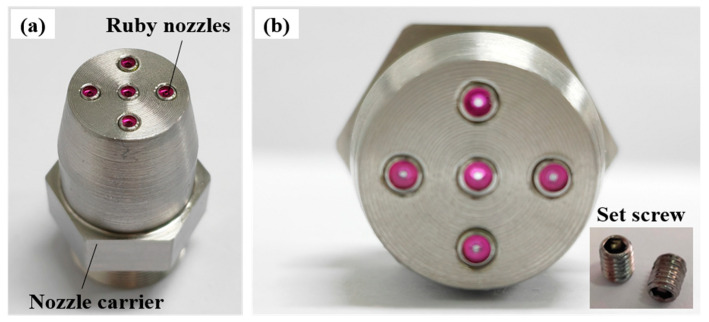
Structure diagram of MJP tool: (**a**) overall structure; (**b**) nozzle partial view.

Two types of multi-nozzle jet polishing tools, linear and cross-distributed, are assessed based on the above simulation results to verify the accuracy of the predictive model for material removal by MJP. The linear nozzle is composed of three single nozzles arranged in a vertical row, while the cross-distributed nozzle is shown in Figure 8, which is composed of two main parts: the ruby nozzle and the nozzle carrier. In Figure 8a, the upper part of the nozzle carrier is connected to the pipeline of the fluid supply system, and the lower part has five threaded holes, so that micro-nozzles made of ruby material can be screwed into the threaded holes according to the actual polishing requirements. Figure 8b shows the installation of the ruby nozzles in a cross configuration. Based on the results of the above simulation, the spacing between the adjacent nozzles is designed to be 5 mm, and the diameter of the nozzle outlet is 0.5 mm. In addition, the current design of the multi-nozzle polishing tool allows for a linear triple-nozzle as well as a single-nozzle arrangement. The threaded holes, which are not required to produce a jet beam, can be replaced by jewelers using hexagon socket recessed set screws to block the fluid. Compared with the existing multi-nozzle polishing tool, the split assembly multi-nozzle polishing tool designed herein is more adaptable, and the nozzle arrangement can be programmed according to the specific needs. Moreover, the nozzle outlet is made of a gemstone material, which is more durable, while the jet beam is more stable than the machined holes, thereby ensuring the stability and uniformity of the material removal from the head of the multi-nozzle polishing tool.

**Figure 9:**
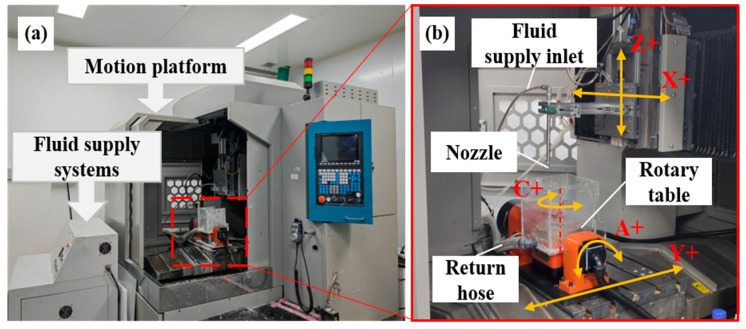
Experimental setup for MJP: (**a**) machine layout; (**b**) motion platform.

Figure 9 shows the MJP process system used in the experiments, which consist of three parts: the motion platform, the circulating fluid supply system, and the nozzle. To ensure the consistency of the initial surface, the quartz glass specimens were subjected to pretreatment using 3 µm and 1 µm diamond grinding fluids prior to the experiment. The grinding time was fixed at 20 min for all samples, ensuring the consistency of the brittle damage characteristics and surface roughness across different specimens. The surface roughness of all samples was approximately 199 nm.

The initial stage is the spot polishing experiment, which aims to validate the accuracy of the predictive model for material removal rates for multi-nozzle jet polishing. Table 2 provides a detailed account of the experimental parameters.

The second stage comprises surface polishing experiments and is conducted with a single nozzle and a linear arrangement of three nozzles for comparison. The process parameters are identical to those outlined in Table 2. In addition, the experiments employed a raster path strategy for surface polishing. Since the nozzle exit spacing was 5 mm, the feed length of the nozzles in the X and Y directions was controlled to be 5 mm. Therefore, the tool traversed a feed length of 5 mm in the main feed direction during each pass and then moved 0.05 mm in the pick-feed direction to commence the subsequent pass. These parameters ensure a systematic and uniform polishing process, covering the entire polishing area effectively. Ultrasonic cleaning was used to clean the sample before and after the polishing process, and at the same time, the drying box was used so that the sample was dried in a drying oven. The polished surface was measured by a 3D optical profilometer, and the weight of the sample glass before and after polishing was measured by an analyzing balance, then the difference between the two values was calculated to obtain the material removal rate (g/min), which was given by the following formula:

(10) $$MRR=\frac{M_{0}-M}{T}$$

where $M_{0}$ is the mass of the sample glass before processing, M is the mass of the sample glass after processing, and T is the processing time.

#### 3.2.2. Results and Discussion

**Figure 10:**
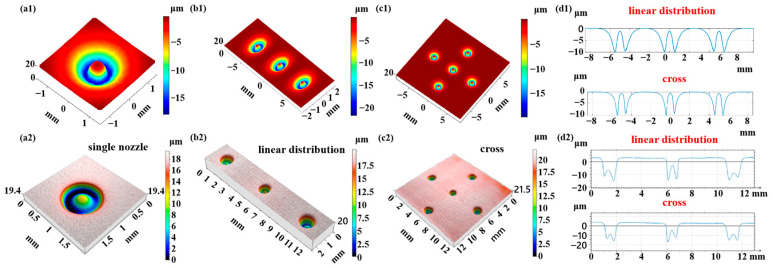
Comparison of simulated and experimental removal profiles under different distribution forms: (**a1**–**d1**) simulated removal profiles; (**a2**–**d2**) experimental removal profiles.

**Figure 11:**
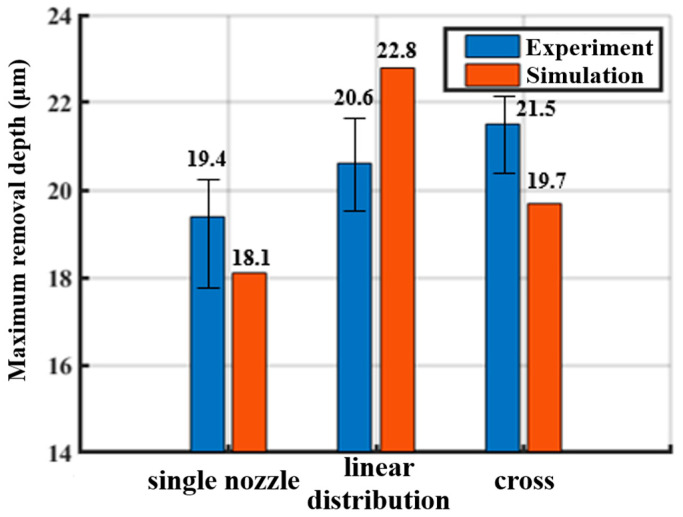
Comparison of experimental and simulated removal depths under different distribution forms.

The experimental results of the single-point removal profiles for multi-nozzle jet polishing under the same process conditions are shown in Figure 10(a2–c2). Compared with the simulation prediction results in Figure 10(a1–c1), it can be found that the removal profiles obtained under the experimental conditions are basically the same as the material removal distribution predicted by the simulation, and the annular removal profiles corresponding to each jet beam have good uniformity. Concurrently, no further material removal resulting from the interference phenomenon can be discerned between the jet beams, thereby substantiating the assertion that the material removal prediction model formulated in this study is capable of accurately predicting the material removal in the complex flow field of the multi-nozzle polishing tool. As shown in Figure 10(d2), the cross-sectional profiles of the removal functions of the nozzle outlets in a straight line and a cross arrangement are intercepted, and it can be observed that the removal depths of each jet outlet are relatively close to each other, which indicates that each jet outlet in the nozzle polishing tool can complete the removal of the material independently, without affecting each other, thereby resulting in the inhomogeneity of the removal of the material. However, it is worth noting that the annular removal profile appears to be deeper on one side, which may be due to the tilting of the nozzle outlets or the uneven surface of the workpiece. In order to further quantitatively analyze the prediction accuracy of the model, Figure 11 shows the experimentally and simulation-predicted maximum removal depths for different nozzle arrangements. For a single nozzle, the experimentally obtained maximum removal depth is 19.4 μm, and the simulation-predicted maximum depth is 18.1 μm, with a prediction error of 7.2%. For three nozzles in a linear arrangement, the experimental and simulation results are 20.6 μm and 22.8 μm, respectively, with a prediction error of 9.6%. For multiple nozzles with cross-cross distribution, the maximum removal depths predicted by experiment and simulation are 21.5 μm and 19.7 μm, respectively, with a prediction error of 9.1%. In conclusion, the material removal prediction model demonstrates a high degree of accuracy, with success rates exceeding 90% across various distribution forms. This outcome substantiates the model’s reliability and predictive capacity.

**Figure 12:**
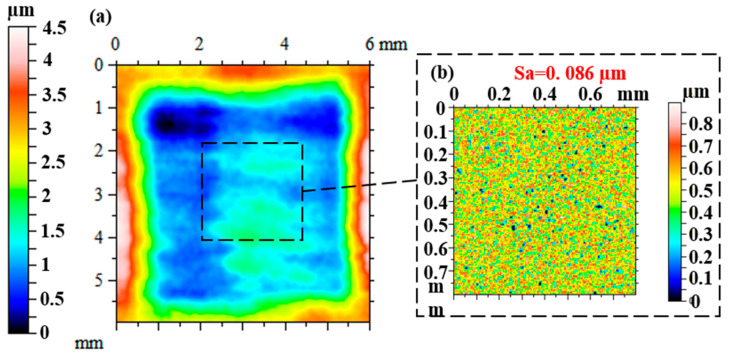
Results of the surface polishing experiment using a single nozzle: (**a**) removal profile; (**b**) microscopic morphology.

**Figure 13:**
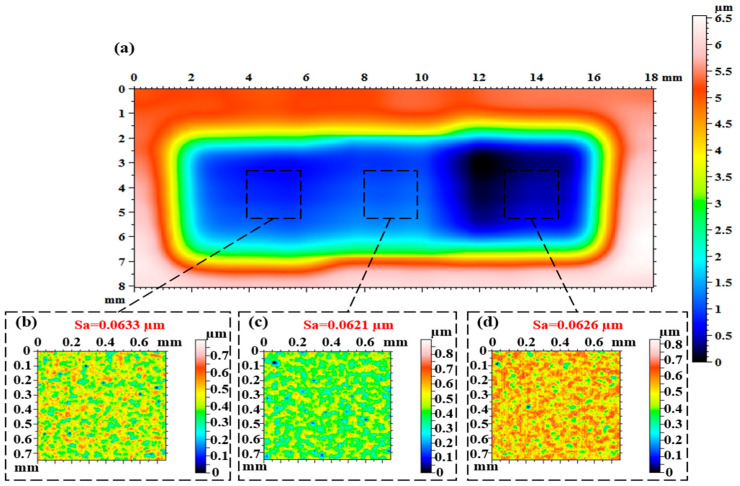
Results of the surface polishing experiment using multiple nozzles: (**a**) removal profile; (**b**) microscopic morphology of region one; (**c**) microscopic morphology of region two; (**d**) microscopic morphology of region three.

Figure 12 and Figure 13 show the workpiece surface after polishing with a single nozzle and a multi-nozzle tool head, respectively, and it can be seen that the polishing area of the multi-nozzle is three times greater than that of the single nozzle for the same polishing time. Since the single nozzle has only one jet beam to remove material from the workpiece surface, the size of the polishing area is the same as the feed length of the nozzle, so the polishing area is 5 × 5 mm^2^. While the linear arrangement of the three nozzles provides three jet beams to polish the target surface at the same time, although the space of the machine movement does not change, the three removal areas are stacked on top of each other, and finally a rectangular removal area is formed, as shown in Figure 13a, with a size of 5 × 15 mm^2^. Comparing Figure 12a and Figure 13a, it can be found that the maximum removal depth of the multiple nozzles is slightly larger than that of the single nozzle, and it was identified that the initial surface shape, the precision of the machine movement, and the change in the concentration of the polishing fluid during the polishing process all have an effect on the amount of material removed. The change in mass after polishing was measured using an analytical balance, and the material removal rates were calculated to be 0.6 × 10^−5^ g/min and 1.9 × 10^−5^ g/min for the single and multiple nozzles, respectively, which is a three-fold increase in the material removal rate for the multi-nozzle design. For the single nozzle, it can be observed that the machined surface is not flat due to the differences in the morphology of the initial surface regions and the uniform polishing preserving the initial surface shape error, as shown in Figure 12a. For multiple nozzles, it can be found that the removal depths corresponding to the three nozzle outlets are not the same, as shown in Figure 13a, with the right nozzle corresponding to a greater removal depth, while the edges of the removed areas do not exactly overlap. It was identified that the poor drilling accuracy of the jewel nozzle outlets and the poor accuracy of the threaded connection between the jewel nozzles and the nozzle carriers could cause the jet beam to be skewed with respect to the surface of the workpiece, resulting in a change in the distribution of the removed material. In addition, the high-frequency microscopic morphology of the machined surfaces was measured, and it can be seen that the microscopic morphologies of the machined areas are basically similar for both single nozzles and multiple nozzles, and the surface roughness Sa is relatively close to that of the machined surfaces. Furthermore, it is noteworthy that the surface roughness (Sa) values of the areas corresponding to the three nozzle outlets of the multi-nozzle polishing tool are 0.0633, 0.0621, and 0.0626 µm, respectively, which means that the micro-material removal process caused by each jet beam is basically the same. The above analyses fully demonstrate that the multi-nozzle structure devised in this paper can markedly enhance the material removal rate during FJP, while simultaneously ensuring optimal processing quality.

## 4. Conclusions

In order to effectively improve the processing efficiency of the jet polishing process, this article offers an integrated theoretical and experimental investigation of the flow field characteristics and particle motion characteristics in MJP, designs and optimizes a multi-nozzle structure with the help of CFD simulation methods, and proves the high efficiency of the multi-nozzle structure using experimental methods. The principal findings are summarized as follows:

CFD simulations showed that for a nozzle outlet diameter of 0.5 mm and spacings of 1, 2, 3 and 5 mm, the interference decreases with increasing outlet spacing. At 5 mm the jets operate independently with no additional material removal due to interference.

When the nozzle outlet spacing is fixed at 5 mm and the diameters are 0.5, 1.0, and 2.0 mm, respectively, the interference between the jet beams becomes progressively more severe as the outlet diameter increases, and a high-pressure region with two sharp ends and a wide center appears between adjacent jet beams, the removal profile is distorted and additional material removal can be observed.

0.5 mm outlet diameter and 5 mm nozzle spacing, the simulated material removal distribution closely matched experimental results, regardless of nozzle configuration, with a maximum prediction error of less than 10%. This confirmed the model’s reliability in forecasting removal profiles for various nozzle structures, enabling simulation-based multi-nozzle design.

The material removal rate of the linear three-nozzles is three times higher than that of the single-nozzles, reaching 1.9 × 10^−5^ g/min and 0.8 × 10^−5^ g/min, respectively, and the surface micromorphology of the two is similar. In addition, the surface roughness Sa of the area corresponding to the three outlets of the linearly distributed multiple nozzles was very close to 0.0633, 0.0621 and 0.0626 µm, respectively.

## Figures and Tables

**Table 1:** Simulation parameters of flow field characteristics in MJP.

Parameters	Settings
Multiphase flow model	VOF
Volumetric	Implicit
Turbulence modelling	Realizable k-ε
Solver	Pressure-based
Solver algorithm	Coupled
Gradient	Least squares cell based

**Table 2:** Experimental parameters for fixed-point polishing.

Experimental Parameters	Settings
Abrasive particles	CeO_2_
Particle diameter (µm)	6
Jet pressure (bar)	10
Target distance (mm)	5
Impact angle (°)	90
Processing time (min)	10
Polishing fluid concentration	1:12 (abrasive to water)

## Data Availability

The data are contained within the article.
